# Microbubbles in the Aorta and Left Ventricle of a Patient with a Left Ventricular Assist Device: A Unique Presentation of Pump Thrombosis Leading to Urgent Surgery

**DOI:** 10.7759/cureus.2463

**Published:** 2018-04-11

**Authors:** Kantha R Kolla, Simon Maltais, Naveen L Pereira, Hari P Chaliki

**Affiliations:** 1 Cardiology, Mayo Clinic, Rochester, MN, USA; 2 Cardiology, Mayo Clinic, Scottsdale, AZ, USA

**Keywords:** left ventricle assist device, pump thrombosis, pump exchange

## Abstract

Microbubble formation occurs due to the cavitation phenomenon. We report a rare echocardiographic finding of microbubbles in a patient’s aorta and left ventricle due to pump thrombosis in the left ventricle assist device, requiring pump exchange surgery.

## Introduction

High power spike alarms were noted on an externally worn pump device in a patient with a left ventricle assist device. Echocardiography showed microbubbles in the aorta and left ventricle. On further evaluation, it was found that these microbubbles had resulted from an obstruction due to thrombus in the left ventricle assist device.

## Case presentation

**Figure 1:**
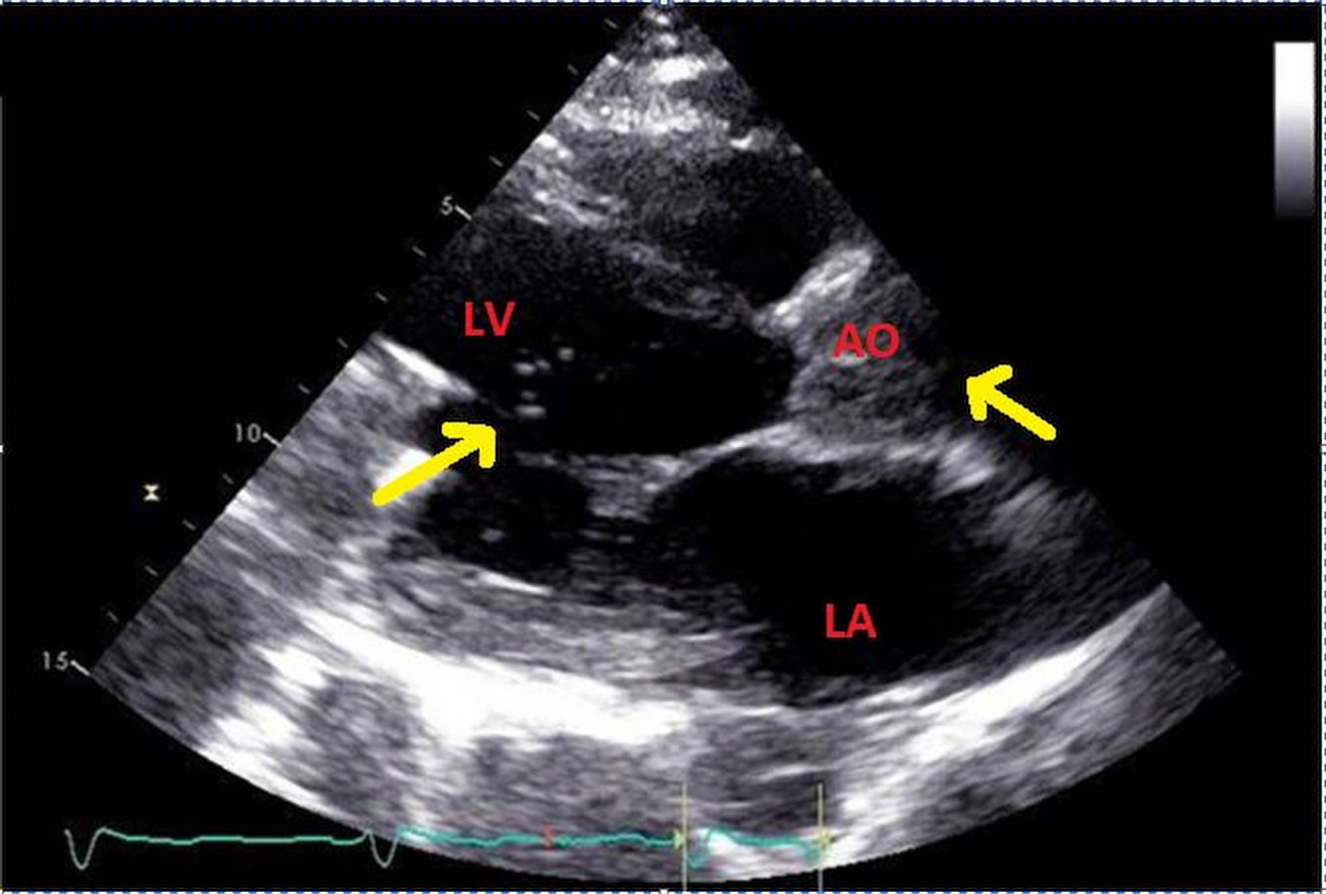
Transthoracic echocardiogram showed microbubbles (arrows) in the aorta and left ventricle LV: left ventricle; AO: aorta; LA: left atrium

**Figure 2:**
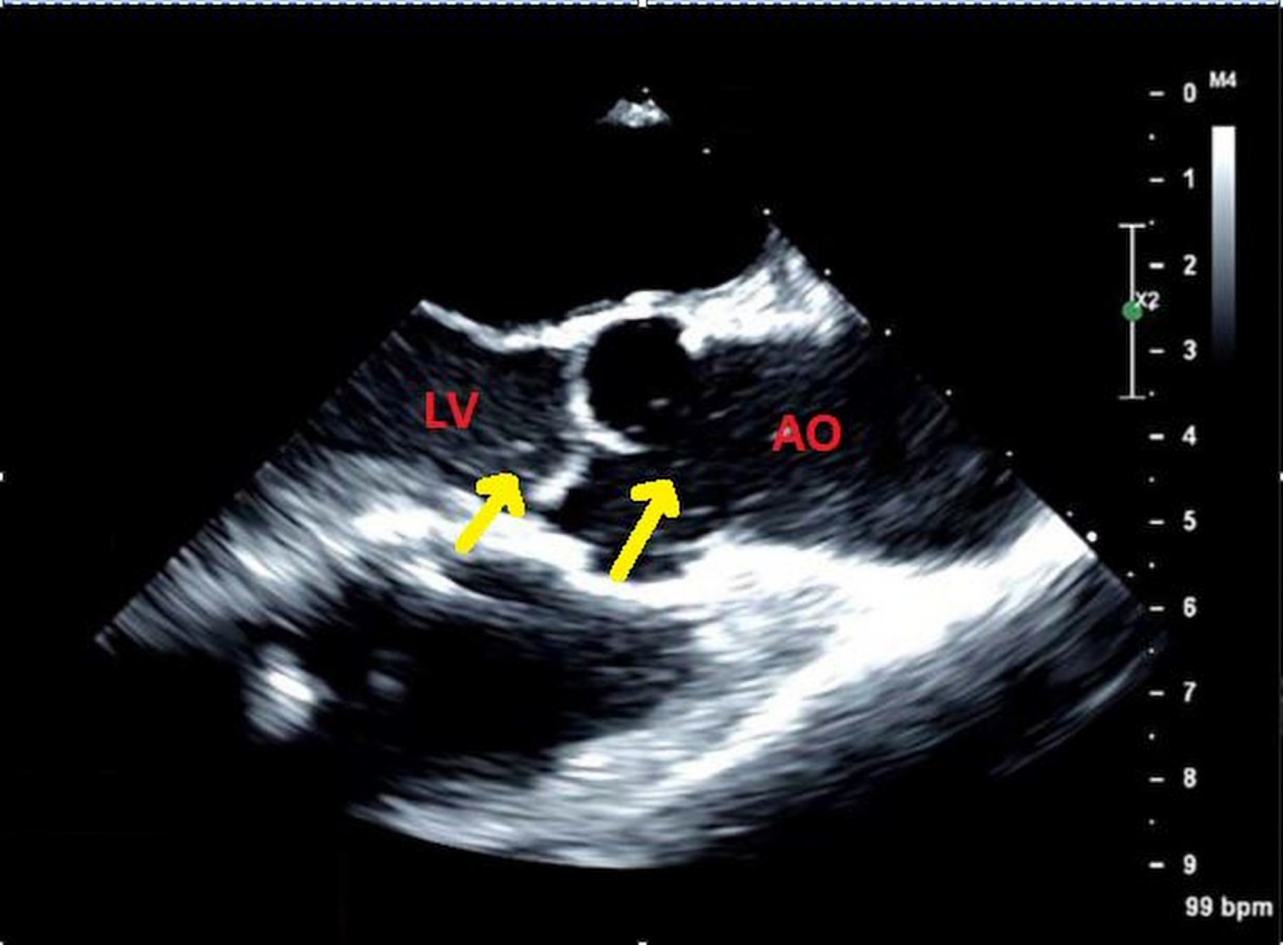
Intraoperative transesophageal echocardiogram before pump exchange showed microbubbles (arrows) in the aorta and left ventricle LV: left ventricle; AO: aorta

A 61-year-old woman with a history of left ventricular assist device (LVAD) pump thrombosis was managed medically for four months before readmission to the hospital with dark urine and high power spikes noted on her device. Laboratory tests showed elevated lactate dehydrogenase (1,621 U/L, from 183 U/L four months earlier) and total bilirubin of 1.6 mg/dL (from 0.3 mg/dL). Transthoracic echocardiography showed microbubbles in the aortic sinus that were entering intermittently into the left ventricle (LV) (Figure 1). Although medical treatment was favored before these findings were known, the observations suggested progressive pump thrombosis and “cavitation.” The patient was emergently transferred to the operating room for pump exchange. Intraoperative transesophageal echocardiography before pump exchange confirmed the presence of microbubbles in the aorta and LV (Figure 2), (Video 1). During surgery, thrombus was noted around the pump rotor and the LVAD was nearly occluded.

**Figure 3:**
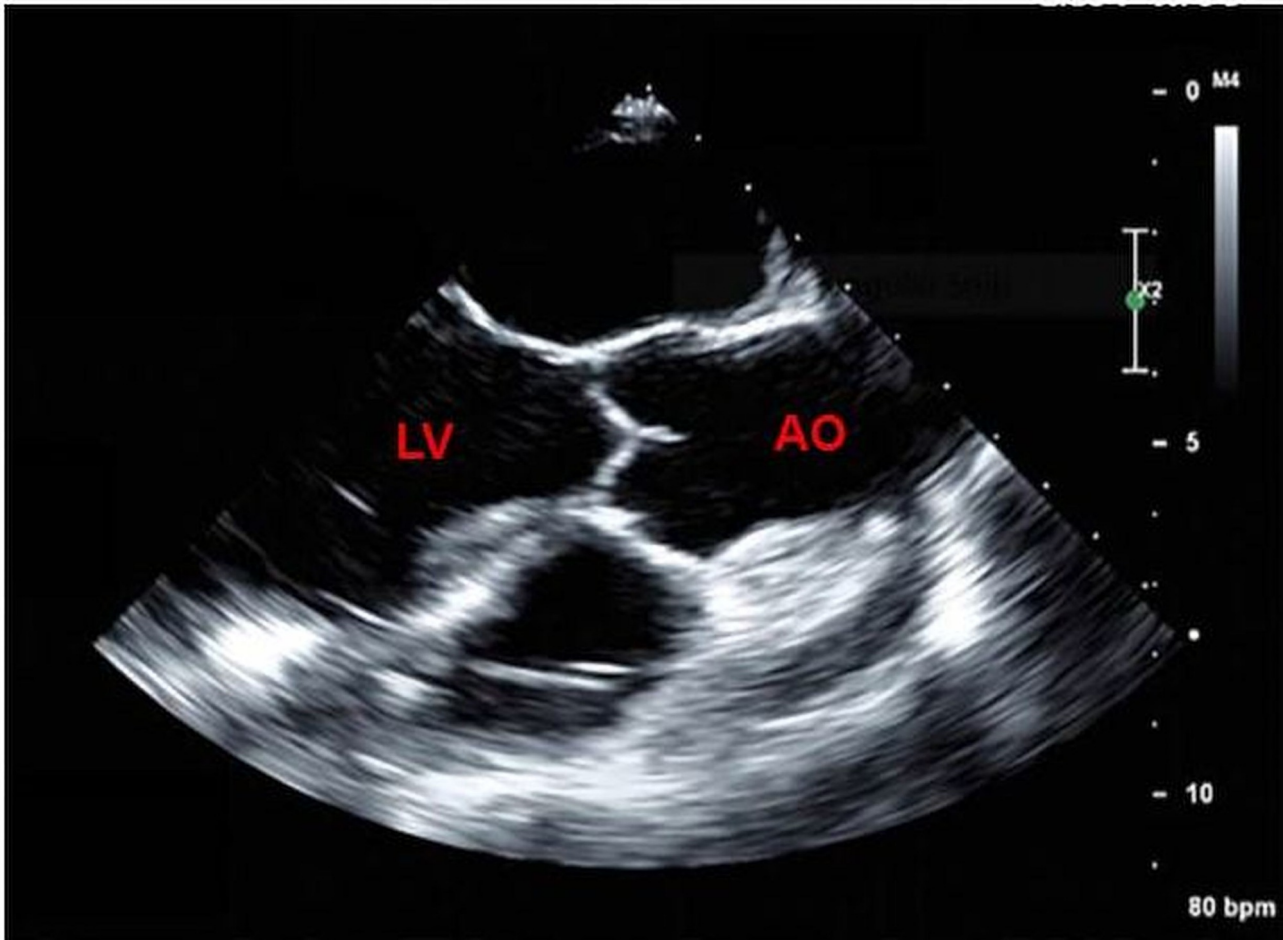
Postoperative transesophageal echocardiogram showed the absence of microbubbles LV: left ventricle; AO: aorta

**Figure 4:**
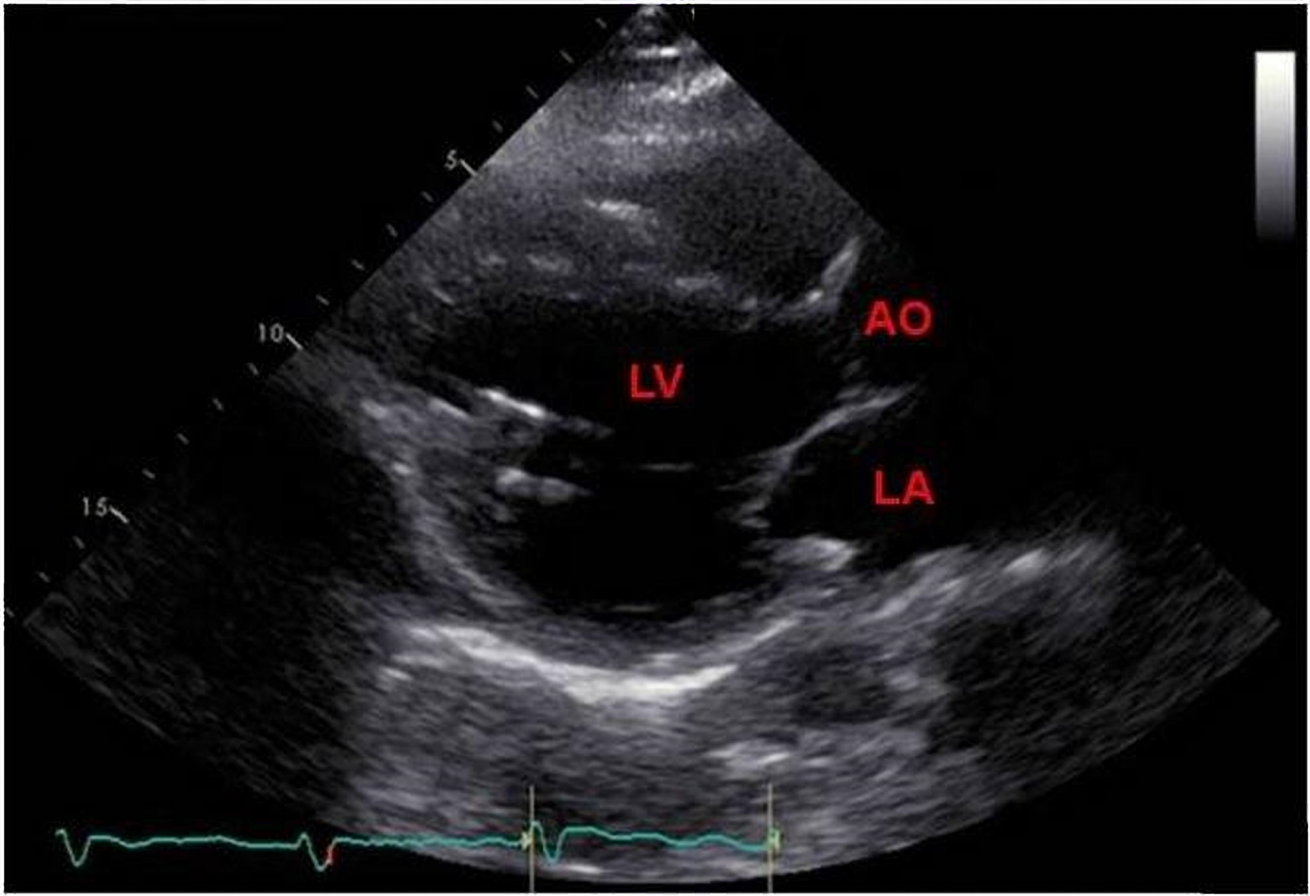
Follow-up transthoracic echocardiography showed the absence of microbubbles in the aorta and left ventricle LV: left ventricle; AO: aorta; LA: left atrium

Transesophageal echocardiography after pump exchange showed a normally functioning LVAD and no microbubbles (Figure 3). Cefepime and vancomycin were initiated for driveline site infection, and aspirin, warfarin, and heparin were initiated postoperatively. Transthoracic echocardiography five days after surgery showed a normally functioning LVAD and no microbubbles in the LV (Figure 4), (Video 2).

**Video 1:** Preoperative echocardiogram showing presence of microbubbles in aorta and left ventricle

**Video 2:** Postoperative echocardiogram showing absence of microbubbles in heart chambers

## Discussion

Retrospective studies of patients with LVAD show an alarming increase in the incidence of pump thrombosis, which was associated with notable morbidity and mortality [1-2]. Echocardiography has a major role in evaluating patients with suspected pump thrombosis. The Bernoulli effect suggests that the obstruction causes a local reduction in pressure at the areas of flow acceleration. The reduced pressure likely causes cavitation from the release of dissolved gases (microbubbles) in the blood [3]. Our patient likely had microbubble formation caused by pressure changes due to LVAD pump thrombosis. Thus, the detection of microbubbles on echocardiography is an indicator of pump thrombosis that should prompt early surgery for pump exchange. These microbubbles are transient but may cause organ injury and neurologic sequelae in rare instances. Thus, this case illustrates the unique finding of microbubbles in the aorta and LV of a patient with LVAD pump thrombosis and hemolysis requiring urgent pump exchange.

## Conclusions

Transesophageal echocardiography showed the unique finding of microbubbles in the aorta and left ventricle. This finding played a pivotal role in changing the treatment plan from medical management to immediate pump exchange surgery and thus helped in providing accurate patient care.
